# Prevalence of Repeat Cesarean Section in a Tertiary Care Hospital

**DOI:** 10.31729/jnma.5375

**Published:** 2020-09-30

**Authors:** Jyotshna Sharma, Sanjeeb Tiwari, Saraswati M Padhye, Bidya Mahato

**Affiliations:** 1 Department of Obstetrics and Gynaecology, Kathmandu Medical College, Sinamangal, Kathmandu, Nepal; 2 Department of General Practice and Emergency Medicine, Maharajgunj Medical Campus, Institute of Medicine, T.U., Maharajgunj, Kathmandu, Nepal

**Keywords:** *birth*, *cesarean section*, *vaginal birth after cesarean*

## Abstract

**Introduction:**

Cesarean section is the surgical delivery of a baby through an incision made in the mother's abdomen and uterus. Repeat cesarean section has recently increased, partly because of concern about increased risk of uterine rupture in women attempting vaginal birth after cesarean delivery. Among the women who underwent cesarean section in their first delivery, 80-96% had a second surgical delivery. Therefore, the present study aimed to describe the prevalence of repeat cesarean section among Nepali women presented at Kathmandu Medical College and Teaching Hospital who had a previous cesarean section.

**Methods:**

This was a descriptive cross-sectional study conducted in Kathmandu Medical College and Teaching Hospital from 1^st^ of February to 31^st^ of May 2020. Ethical approval was taken from the Institutional Review Committee of the Kathmandu Medical College. Convenient sampling was done. All pregnant patients between gestational ages of 37-40 weeks with previous cesarean section admitted for safe confinement were included in the study.

**Results:**

Among the 104 women, who had prior cesarean section, 99 (95.19%) had second cesarean section and 5 (4.81%) had vaginal birth after cesarean. The most common indication for the first cesarean section was fetal distress 31 (29.81%) while the indication for the second cesarean section among previously cesarean section women was cephalo pelvic disproportion 39 (39.40%).

**Conclusions:**

The proportion of cesarean section in both first and subsequent delivery is quite high. This high rate may compromise the reproductive future of the women who underwent consecutive cesarean section with possible consequent complications.

## INTRODUCTION

The incidence of cesarean section is increasing. The contributory factors are decrease training for the clinician in instrumental vaginal delivery, medico-legal issues, the increasing use of electronic fetal heart rate monitoring in labor, and maternal request for cesarean delivery.^1–3^ Repeat cesarean section (C-section) after a previous cesarean section has been a significant contributor to overall increased C-section rate and accounts for more than one-third of all Cesarean deliveries all around the world.^4^

The medical profession has been concerned about the risk of catastrophic uterine rupture for women with previous delivery by cesarean section.^5–7^ Studies have shown that mother and fetus may be at greater risk than previously thought because uterine rupture has stirred controversy about the safety of vaginal birth after cesarean (VBAC). Both repeat cesarean section and VBAC have benefits and risks for pregnant women.^8–10^

This study aimed to describe the prevalence repeat cesarean section among Nepali women who had a previous cesarean section.

## METHODS

This is a descriptive cross-sectional study conducted from the 1^st^ of February to 31^st^ of May 2020 (four months period) at Kathmandu Medical College and Teaching Hospital in all the pregnant women with the previous cesarean section. Ethical approval was obtained from the Institutional Review Committee of Kathmandu Medical College. All the pregnant women between gestational ages of 37-40 weeks who were admitted at Kathmandu Medical College and Teaching Hospital with a previous cesarean section for safe confinement were included in the study. All pregnant women with previously normal delivery or first-time delivery whether a cesarean section or normal were excluded from the study.

Informed consent was taken from the participants. Detailed clinical history was taken and filled in performa. The convenient sampling method was used. All the data were entered and the Statistical package of the social science (SPSS) version 20.

The sample size was calculated by using formula,

$$\begin{array}{ll} \text{n} & \text{= }{\text{Z}}^{\text{2}}\times \text{p}\times \text{q}/{\text{e}}^{\text{2}}\\ & \text{= }{\left(1.96\right)}^{\text{2}}\times 0.5\times (1-0.5)/{\left(0.1\right)}^{\text{2}}\\ & \text{= }96 \end{array}$$

Where,

n = sample size
Z = 1.96 at 95% Confidence Interval
p = prevalence, 50%
q = 1-p
e = margin of error, 10%

Although the sample size calculated was 96, taking the non-response rate 8%, the total participants included were 104. Once the patients were admitted for confinement, mode of delivery, fetal and maternal complications, and the indication of the prior section was noted. Although, indication of primary and second cesarean section was noted, there is no relation between the indication of cesarean section in same woman as primary cesarean section indication can differ from the indication of second cesarean section.

## RESULTS

From our study, the age of the patient with the previous cesarean range from 19 years to 38 years. The mean age was 29.46 years with a standard deviation of 4.02 years. Out of total pregnancies with the history of the previous cesarean section, 5 (4.80%) had a vaginal birth after cesarean section, and 99 (95.19%) were repeat cesarean section.

All the women were asked for desire mode of delivery, about 73 (70.19%) of women wanted repeat cesarean section, didn't want the trial of labor, while 21 (20.19%) of women were not sure and knowing in detail about the trial of labor in a previous cesarean section then they wanted to undergo repeat cesarean section. However, 10 (9.61%) of women want the trial of labor, but after knowing the failure of labor and limitation in the use of oxytocin 4 (3.84%) choose the repeat cesarean section. Only 6 (5.76%) of women wanted a trial of labor, these women were gravid three or more and had a history of normal delivery at first.

Among 99 repeat cesarean section, 33 (33.33%) underwent emergency cesarean section and 66 (66.66%) were planned cesarean section. There were no significant differences in complications. There was no major complication while 4.04% had a minor complication (blood transfusion and wound infection). In both groups had minor complications, 3 (3.03%) had a blood transfusion after cesarean section and 1 (1.01 %) had wound infection underwent resuturing.

Among the total patients with the previous cesarean section, the indication for the first cesarean section was most commonly fetal distress 31 (29.81%), followed by failed induction 23 (22.12%), cephalo pelvic disproportion 16 (15.38%), malpresentation 11 (10.58%) and others (Table 1).

**Table 1:** Indication of primary section (n = 104).

Variables	n (%)
Fetal distress	31 (29.81)
Failed induction	23 (22.12)
Cephalo pelvic disproportion	16 (15.38)
Malpresentation	11 (10.58)
Intrauterine fetal growth restriction	6 (5.77)
Cesarean-section on maternal request	6 (5.77)
Obstructed labor	5 (4.81)
Multiple pregnancies	4 (3.84)
Bad obstetrics history	2 (1.92)
Total	104 (100)

Out of the total women with the repeat cesarean section, the most common indication for the cesarean section was cephalo pelvic disproportion 39 (39.40%), followed by fetal distress 26 (26.26%), prelabour rupture of membrane 12 (12.12%), malpresentation 8 (8.08%) and others (Table 2).

**Table 2:** Indication of second cesarean section (n = 99).

Variables	n (%)
Cephalo pelvic disproportion	39 (39.40)
Fetal distress	26 (26.26)
Prelabour rupture of membrane	12 (12.12)
Malpresentation	8 (8.08)
Multiple pregnancies	5 (5.05)
Bad obstetric history	5 (5.05)
Antepartum hemorrhage	4 (4.04)
Total	99 (100)

## DISCUSSION

In our study, the total sample size was 104. The age of the patient with the previous cesarean range from 19 years to 38 years. The mean age was 29.46 years with a standard deviation of 4.02 years. The total number of repeat cesarean section was 99 (95.19%). Thus the prevalence of the repeat cesarean section was 95.19% which is quite high. This is similar to the study carried out by Mascarello, et al. which also had a high prevalence of 87.44% repeat cesarean section in their study.^11^ The reason for the repeat cesarean section was mostly cephalo pelvic disproportion 39 (39.40%), fetal distress 26 (26.26%), and pre-labor rupture of membrane 12 (12.12%) in our study. However, the reason for primary cesarean was quite different as a comparison to the repeat cesarean section such as fetal distress 31 (29.81%) was most common followed by failed induction 23 (22.12%) and cephalopelvic disproportion 16 (15.38%) in our study. The study by Cheng, et al. have stated that about one-third cause of cesarean section was previous cesarean section^4^ while in our study 29.46% cesarean section was repeat cesarean section. In our study, only 5 out of 104 had a vaginal birth after cesarean section (VBAC) which is 4.80%.

In the study carried out by McMahon, et al. about 1.3% had major complications and 6.9% had minor complications (puerperal fever, blood transfusion, and wound infection).^5^ While in our study there was no major complication and 4.04% had a minor complication (blood transfusion and wound infection).

This study was conducted in the tertiary care hospital among the limited number of the population which cannot be generalized to the whole population with the different hospital settings. There may be seasonal variation as the study was a descriptive cross-sectional study.

## CONCLUSIONS

In our study, the proportion of cesarean section in both first and subsequent delivery was quite high. This study has clearly shown that the prior cesarean section is the major indication of repeat cesarean section. Most of the women didn't want a trial of labor which has significantly increased in the rate of repeat cesarean section. Although in our study the complications in the repeat cesarean section are quite less, the complete care should be taken by the individual and clinician level for the repeat cesarean section.

## Conflict of Interest

**None.**
